# *Schisandra chinensis* Bee Pollen Extract Alleviates Obesity by Modulating Gut Microbiota-Driven Bile Acid Metabolism

**DOI:** 10.3390/nu17223597

**Published:** 2025-11-18

**Authors:** Xin An, Jingxuan Zhang, Runwen Chou, Cheng Zhao, Haoan Zhao, Wei Cao, Ni Cheng

**Affiliations:** 1 College of Food Science and Technology, Northwest University, Xi’an 710069, China; 2 Bee Product Research Center of Shaanxi Province, Xi’an 710065, China

**Keywords:** *Schisandra chinensis* bee pollen extract (SCPE), obesity, gut microbiota, bile acid (BA)

## Abstract

Background: Bee pollen is a uniquely complete nutritional product that has shown promise in alleviating obesity. While existing research has largely focused on the role of gut microbiota in obesity, the mechanisms by which bee pollen influences bile acid (BA) metabolism via microbial regulation remain poorly understood. Methods: This study hypothesized that *Schisandra chinensis* bee pollen extract (SCPE) could mitigate high-fat diet (HFD)-induced obesity by regulating BA metabolism. Results: In a 12-week animal experiment, SCPE supplementation significantly reduced body weight gain, lipid accumulation, and adipocyte hypertrophy, while improving insulin sensitivity and relieving hepatic oxidative stress. These benefits were attributed to an increased relative abundance of bile salt hydrolase (BSH)-producing microbes, including *Bacteroides*, *Lachnospiraceae NK4A136 group*, and *Akkermansia*, which modulated BA metabolism by improving the expression of BA metabolism-related genes and reducing the concentrations of various types of BAs. Conclusions: These findings provide new insights into the mechanism by which SCPE alleviates obesity through the gut microbiota-BA axis and support the potential of bee pollen as a functional food for obesity management.

## 1. Introduction

Obesity, a chronic disease driven by metabolic dysregulation, has emerged as a major global public health challenge [1]. Beyond the abnormal lipid accumulation resulting from imbalances in energy metabolism, obesity is closely associated with key pathological processes such as intestinal barrier dysfunction, systemic chronic inflammation, and oxidative stress [2]. Notably, obesity shares a significant synergistic pathogenic relationship with various metabolic diseases, including type 2 diabetes, non-alcoholic fatty liver disease (NAFLD), and certain cancers [3]. Understanding the underlying mechanisms and exploring effective intervention strategies have therefore become key areas of current research.

In recent years, the gut microbiota, widely regarded as the ‘second genome’, has garnered increasingly recognized for its pivotal role in metabolic regulation. Studies have demonstrated that obesity phenotypes can be transferred between individuals via fecal microbiota transplantation (FMT) in germ-free animals [4], and that transplanting microbiota from lean donors to obese recipients can reverse metabolic abnormalities [5]. Further research suggests that gut microbes influence energy metabolism through multiple pathways, including modulating nutrient and energy absorption [6], improving insulin resistance [7], inhibiting fat and cholesterol synthesis [8], maintaining intestinal barrier integrity and gut immunity [9], reducing pro-inflammatory cytokine secretion [10], and alleviating oxidative stress [11]. However, the specific molecular mechanisms through which the gut microbiota contributes to these processes remain to be fully elucidated.

As amphipathic molecules, BAs are synthesized from cholesterol in the liver and subsequently metabolized by the gut microbiota, playing an integral role in maintaining cholesterol homeostasis through their tightly regulated synthesis and excretion [12,13]. Accumulating evidence indicates that HFD impairs BA metabolism, thereby disrupting energy homeostasis and contributing to various chronic metabolic diseases [14]. The gut microbiota is indispensable for converting primary BAs into secondary BAs through processes such as deconjugation and 7-dehydroxylation, which are crucial for maintaining systemic cholesterol homeostasis and regulating glucose metabolism [15]. Recent studies highlight that specific gut microbes, such as *Akkermansia* and *Bacteroides*, influence lipid metabolism by modulating BA metabolic profiles [16,17]. These findings indicate that functional food components may exhibit anti-obesity properties by targeting the “gut microbiota-BA” axis.

Bee pollen is widely recognized as a “complete food” owing to its rich nutritional components. *S. chinensis* bee pollen (SCP), a natural product collected by bees from *S. chinensis* plants, combines the nutritional benefits of bee pollen with the medicinal properties of *S. chinensis*. As a functional food, SCP has attracted considerable attention for its antioxidant, anticancer, and gut barrier-enhancing effects [18,19]. Our previous work demonstrated that the polyphenol-rich extract from SCP (SCPE) contains 1.89 mg/g of naringenin and 101.83 mg gallic acid equivalents (GAE) /g of total phenolics, and that SCPE ameliorates dextran sulfate sodium (DSS)-induced colitis in C57BL/6 mice by reshaping the gut microbiota [20]. More importantly, SCPE was also found to alleviate NAFLD and obesity in HFD-induced obese mice, with accompanying alterations in gut microbiota composition [21]. To further investigate the interplay between gut microbiota and BA metabolism in this process, we conducted FMT combined with targeted metabolomics to analyze changes in the BA profiles. Specifically, this study aimed to address the following questions: (1) Can SCPE-regulated microbiota transfer its anti-obesity effects? (2) Does SCPE ameliorate obesity by remodeling the BA metabolism? (3) How does the “microbiota-BA” interaction influence gut-liver axis function? Our findings offer new theoretical insights into how SCPE targets the “gut microbiota-BA” metabolism axis.

## 2. Materials and Methods

### 2.1. Preparation of SCPE

The SCP used in this study was collected during the flowering season of *S. chinensis* (July–August 2021) from a local apiary in Xianning, Hubei Province, China (29°36′ N, 114°28′ E) [21]. To obtain a pure and botanically homogeneous sample, pollen traps were installed at the entrances of ten beehives for bee pollen collection. Few flowering plants were present in the surrounding area. Approximately 5 kg of fresh bee pollen was obtained in total, pooled, thoroughly mixed to ensure homogeneity, and stored at −20 °C until subsequent analysis. The botanical origin of the pollen was confirmed by palynological identification. SCPE was obtained according to the technique outlined in our prior study [20]. Briefly, the bee pollen was mixed with 75% ethanol at a 1:10 (*w*/*v*) ratio and subjected to reflux extraction at 75 °C for 2 h. The resulting solution was centrifuged (4800× *g*, 10 min), and the supernatant was collected and vacuum-concentrated to yield a polyphenol-rich SCPE.

### 2.2. Animals and Experimental Design

Male C57BL/6 mice (6 weeks old, 20.0 ± 1.0 g) were supplied by the laboratory animal research center of Fourth Military Medical University (license number: SCXK (Army) 2019-001). The normal chow diet (ND; 3.6 kcal/g, 10% of energy from fat; D12450B) and HFD (4.5 kcal/g, 45% of energy from fat; D12251) were obtained from Guangdong Provincial Medical Laboratory Animal Center (Guangzhou, China). The mice were housed under controlled environmental conditions (23 ± 1 °C, 50 ± 5% relative humidity, 12 h light/dark cycle) with free access to food and water. All experimental protocols were reviewed by the Ethics Review Board of the Laboratory Animal Center at Northwest University (Permit Code: NWU-AWC-20220907M) and strictly adhered to the National Institutes of Health Guidelines Guide for the Care and Use of Laboratory Animals.

Following a week of acclimation, mice were randomly divided into 2 groups: the ND group (*n* = 10), receiving a standard chow formulation, and the HFD group (*n* = 30), provided with an HFD. After 6 weeks of feeding, the HFD-fed mice were further randomized into 3 subgroups: (1) HFD group (*n* = 10), continued on the HFD and received a daily saline gavage; (2) HFD + SCPE group (HE group, *n* = 10), continued on the HFD and received a daily gavage of SCPE (20.4 g/kg BW); (3) HFD + FMT group (FMT group, *n* = 10), continued on the HFD and received a daily gavage of fecal microbiota (100 μL) derived from the HE group donor mice. SCPE and FMT treatments were administered for 10 weeks, with the total experimental duration spanning 16 weeks. Body weight and food intake were monitored biweekly throughout the study. The FMT protocol was adapted from a previously described method [10]. Fresh fecal samples from HE group donor mice were homogenized in five volumes of sterile saline, vortexed, and centrifuged (800× *g*, 3 min, 4 °C) to collect the supernatant for transplantation. Prior to FMT, recipient mice were gavaged with 20 mg/mL vancomycin (0.1 mL/30 g BW) in saline and provided drinking water containing a 200 mg/mL compound polyethylene glycol solution for 3 days to deplete their gut microbiota. At the end of the treatment, mice were fasted for 12 h, anesthetized with pentobarbital, and blood was collected via enucleation of the eyeballs, followed by euthanasia by cervical dislocation. Blood samples were centrifuged (3000× *g*, 10 min, 4 °C) to obtain serum. The liver and epididymal fat were dissected, rinsed with saline, and weighed, with portions fixed in 4% paraformaldehyde and the remaining samples rapidly frozen in liquid nitrogen for further analysis. Colon tissues and fecal samples were collected and stored at −80 °C until subsequent analysis.

### 2.3. Biochemical Analysis

Commercial kits (Nanjing Jiancheng Bioengineering Institute, Nanjing, China) were used to quantify aspartate aminotransferase (AST), alanine aminotransferase (ALT), triglyceride (TG), total cholesterol (TC), high-density lipoprotein cholesterol (HDL-C), low-density lipoprotein cholesterol (LDL-C), and total bile acid (TBA) in the serum; TG, TC, malonaldehyde (MDA), glutathione peroxidase (GSH-Px), superoxide dismutase (SOD), and TBA in the liver; and TBA contents in the feces.

### 2.4. Histological Characterization

Liver and epididymal adipose tissues were preserved in 4% paraformaldehyde for 24 h, paraffin-embedded, and sectioned for histological analysis. Liver tissue was stained with Hematoxylin and eosin (H&E) and Oil Red O, while epididymal adipose tissue was stained with H&E. Images were captured using a Nikon Eclipse Ci-L optical microscope (Nikon Corporation, Tokyo, Japan) at 200× magnification.

### 2.5. Oral Glucose Tolerance Test (OGTT)

Following a 15-week experimental period, mice were fasted overnight and then gavaged with a 50% glucose solution (2 g/kg body weight; Aladdin, Shanghai, China). Blood glucose levels were monitored at 30 min intervals following administration, and the area under the curve (AUC) was subsequently calculated.

### 2.6. Quantitative Real-Time PCR (qRT-PCR) Analysis

Total RNA was extracted from liver and colon samples using the miniBEST Universal RNA Extraction Kit (TaKaRa, Dalian, China). Subsequently, cDNA was synthesized using the PrimeScript RT Master Mix (TaKaRa), following the manufacturer’s instructions. qRT-PCR was conducted on the LightCycler 480 system (Roche, Basel, Switzerland) as described in a previous study [20]. Relative gene expression levels were quantified using the 2^−ΔΔCt^ method, with *Gapdh* serving as the internal control gene. Primer sequences used in this study are listed in Table S1.

### 2.7. Gut Microbiota Analysis

16S rRNA gene sequencing was carried out as described in our previously described method [22]. Operational taxonomic unit (OTU) analysis was performed to evaluate biodiversity indices (species richness/evenness), whereas principal component analysis (PCA) was used to assess structural variations in microbial communities among experimental groups. Significant inter-group differences in species composition were identified using t-tests.

### 2.8. BA Metabolomic Analysis

Fecal BA profiles were quantified using a previously described method [23]. In brief, feces (50 mg) were mixed with methanol (400 μL), sonicated (30 min) and centrifuged (12,000× *g*, 10 min, 4 °C). The resulting supernatant (300 µL) was combined with distilled water (600 μL), and analyzed by UPLC-MS/MS (Agilent Technologies, Santa Clara, CA, USA). The analytical system comprised an Agilent 1290 UPLC coupled with an Agilent 6470 triple quadrupole mass spectrometer. An aliquot (1 µL) was injected and separated on a ZORBAX Eclipse Plus C18 column (2.1 × 100 mm, 1.8 μm, Agilent Technologies, Santa Clara, CA, USA) at 45 °C.

### 2.9. Statistics Analysis

Statistical analyses were performed using SPSS 20.0 and GraphPad Prism (version 7.0). Each group initially included ten mice, and outliers were identified using Grubbs’ test, with data points having *G*_(calculated)_ > *G*_(critical)_ excluded from the analysis. After outlier removal, at least six biological replicates (*n* ≥ 6) per group were retained for the final analysis. The excluded data points were randomly distributed across groups and did not show consistent patterns. Data are presented as means ± standard error of the mean (SEM). Significant differences (*p* < 0.05) were analyzed using One-way analysis of variance (ANOVA), with Duncan’s multiple-range test for post hoc comparisons.

## 3. Results

### 3.1. SCPE Regulates the Gut Microbiota to Alleviate HFD-Induced Obesity and Metabolic Disturbances

**Figure 1:**
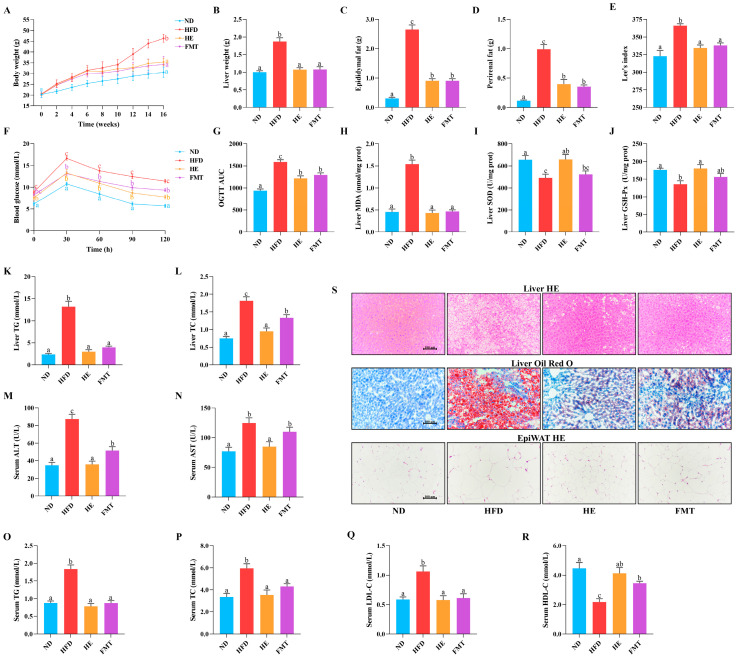
Effect of SCPE and FMT on obesity characteristics in HFD-induced mice. (**A**) body weight, (**B**) liver weigh, (**C**) epididymal weight, (**D**) perirenal weight, (**E**) Lee’s index, (**F**,**G**) OGTT and the corresponding area under the curve (AUC), (**H**) liver MDA, (**I**) liver SOD, (**J**) liver GSH-Px, (**K**) liver TG, (**L**) liver TC, (**M**) serum ALT, (**N**) serum AST, (**O**) serum TG, (**P**) serum TC, (**Q**) serum LDL-C, (**R**) serum HDL-C and (**S**) morphological changes in the liver and epididymis adipose tissue (×200). The different lowercase letters indicate a significant difference (*p* < 0.05) among different groups. The data are presented as the mean ± SEM (*n* ≥ 6).

Following 6 weeks of HFD intervention, HFD-fed mice exhibited a 20% increase in body weight relative to the ND group (Figure 1A), confirming the successful induction of obesity. Typically, HFD-induced obesity is characterized by excessive fat deposition. Herein, we identified significant increases in weights of liver, perirenal fat, epididymal fat, and Lee’s index in the HFD group (Figure 1B–E). Notably, SCPE intervention effectively reversed these changes, with the FMT group exhibiting comparable metabolic improvements. However, the average daily food intake did not exhibit notable variation between the groups (Figure S1A), indicating that the anti-obesity effects of SCPE are independent of appetite regulation. Additionally, HFD-induced obesity is often connected to impaired glucose metabolism. Here, OGTT was performed to assess the impact of SCPE on glucose regulation. The results revealed that fasting blood glucose levels were notably elevated in the HFD group, whereas SCPE and FMT interventions significantly reduced the AUC (Figure 1F,G), indicating that SCPE improves glucose metabolism, potentially through gut microbiota regulation.

HFD-induced metabolic disturbances were associated with a significant imbalance in hepatic oxidative stress. Compared to the ND group, long-term HFD dramatically increased the liver MDA levels and suppressed the enzymatic activities of GSH-Px and SOD (Figure 1H–J). In contrast, SCPE intervention significantly reversed these changes. However, the FMT group only exhibited a marked decrease in MDA levels, suggesting that the polyphenols in SCPE may directly regulate the activities of antioxidant enzymes, an effect that could not be effectively transmitted through FMT. Additionally, we evaluated the role of SCPE in modulating lipid metabolism, as depicted in Figure 1K–R. HFD significantly elevated the serum levels of TG, TC, LDL-C, AST, and ALT, while decreasing HDL-C levels. Furthermore, the hepatic TG and TC levels were also markedly elevated. SCPE intervention effectively reversed these alterations, restoring levels to those comparable to the ND group. Surprisingly, FMT intervention also notably reduced serum TG, TC, LDL-C, and ALT levels, increased HDL-C levels, and decreased hepatic TG and TC accumulation.

H&E and Oil Red O staining were performed for histological examination of the liver and epididymal fat (Figure 1S). Long-term HFD induced hepatocyte hypertrophy, fatty degeneration, inflammatory cell infiltration, and the accumulation of numerous stained lipid droplets. Both SCPE and FMT interventions significantly reduced hepatocyte vacuolization, alleviated inflammatory cell infiltration, and significantly decreased the number and distribution of stained lipid droplets. Furthermore, H&E staining of epididymal fat revealed a marked reduction in both the density and size of adipocytes in the HE and FMT groups, which was aligned with the changes observed in epididymal fat weight.

### 3.2. SCPE Regulates the Gut Microbiota to Improve the Expression of BA Metabolism-Related Genes

**Figure 2:**
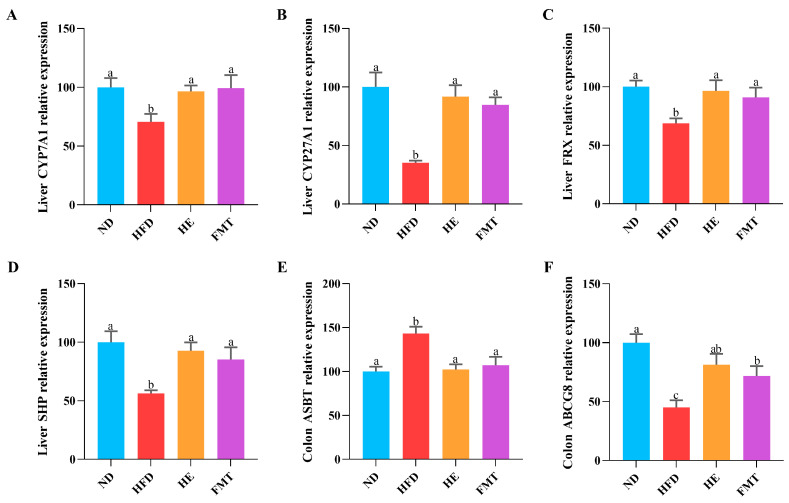
Effects of SCPE and FMT on BA metabolism-related gene expressions in the liver and colon. (**A**) Liver CYP7A1, (**B**) Liver CYP27A1, (**C**) Liver FXR, (**D**) Liver SHP, (**E**) Colon ABST, and (**F**) Colon ABCG8. The different lowercase letters indicate a significant difference (*p* < 0.05) among different groups. The data are presented as the mean ± SEM (*n* ≥ 6).

To further elucidate the potential mechanisms by which SCPE inhibits HFD-induced excessive lipid accumulation, we assessed the expression of genes involved in BA metabolism using qRT-PCR. As showed in Figure 2A–F, the expression of key rate-limiting enzymes in BA synthesis, cholesterol 7α-hydroxylase (CYP7A1) and cholesterol 27α-hydroxylase (CYP27A1), was significantly reduced in the HFD group, along with a marked downregulation of the BA receptor farnesoid X receptor (FXR) and its downstream target gene small heterodimer partner (SHP). In contrast, both SCPE and FMT treatments effectively restored the expression of these genes. Additionally, relative to the HFD group, both the HE and FMT groups exhibited notably increased expression of the BA efflux transporter ATP-binding cassette sub-family G member 8 (ABCG8) and decreased expression of the apical sodium-dependent BA transporter (ASBT) in the colon. These findings suggest that SCPE-modulated gut microbiota may alleviate HFD-induced BA metabolic disturbances and excessive lipid accumulation by regulating the expression of genes related to BA synthesis and transport.

### 3.3. SCPE Reversed HFD-Induced Gut Microbiota Dysbiosis

**Figure 3:**
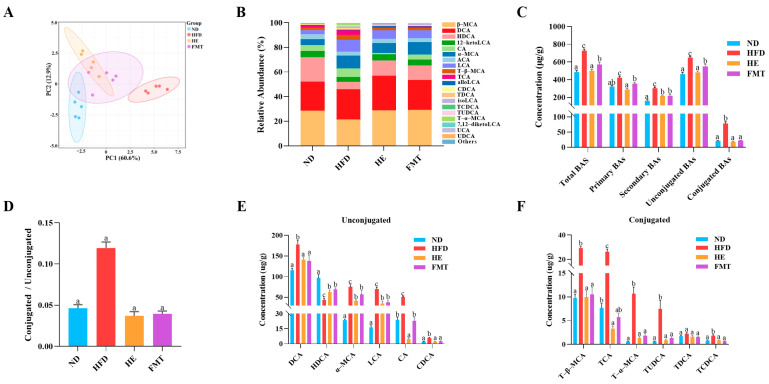
Effects of SCPE and FMT on fecal BA levels in HFD-induced mice. (**A**) PCA scores plot, (**B**) composition of BA profiles, (**C**) proportion of different types of BAs, (**D**) ratio of conjugated to unconjugated BAs, (**E**) contents of different types of unconjugated BAs, and (**F**) contents of different types of conjugated BAs. The different lowercase letters indicate a significant difference (*p* < 0.05) among different groups. The data are presented as the mean ± SEM (*n* = 5).

BAs, as essential signaling molecules, influence metabolic syndrome through dynamic changes in their composition and concentration, which are closely associated with systemic homeostatic imbalances [24]. As illustrated in Table 1, treatment with SCPE and FMT significantly alleviated the HFD-induced increase in serum and fecal TBA contents, whereas no significant differences were observed in hepatic TBA contents among the groups (*p* > 0.05). To further explore the specific alterations in BA profiles, targeted metabolomic analysis was performed on fecal samples collected after 10 weeks of SCPE and FMT intervention using UPLC-MS/MS. PCA revealed a clear separation among the ND, HFD, HE, and FMT groups (Figure 3A). The predominant BAs across all groups were β-muricholic acid (β-MCA), deoxycholic acid (DCA), hyodeoxycholic acid (HDCA), 12-ketolithocholic acid (12-ketoLCA), cholic acid (CA), and α-MCA, which collectively accounted for over 70% of the TBA (Figure 3B). Interestingly, compared to the HFD group, both SCPE and FMT treatments significantly reduced the contents of various BAs (Figure 3C), accompanied by a marked decrease in the ratio of conjugated to unconjugated BAs (Figure 3D). Specifically, for unconjugated BAs, SCPE significantly reduced the contents of DCA, CA, α-MCA, lithocholic acid (LCA), and chenodeoxycholic acid (CDCA), while increasing the content of HDCA (Figure 3E), with similar trends observed in the FMT group. In terms of conjugated BAs, the HFD group exhibited significantly elevated contents of tauro-β-muricholic acid (T-β-MCA), taurocholate acid (TCA), taurochenodeoxycholic acid (TCDCA), tauroursodeoxycholic acid (TUDCA), and T-α-MCA, which were effectively reversed by SCPE and FMT interventions (Figure 3F).

### 3.4. SCPE Regulates the Gut Microbiota to Optimize BA Composition

**Figure 4:**
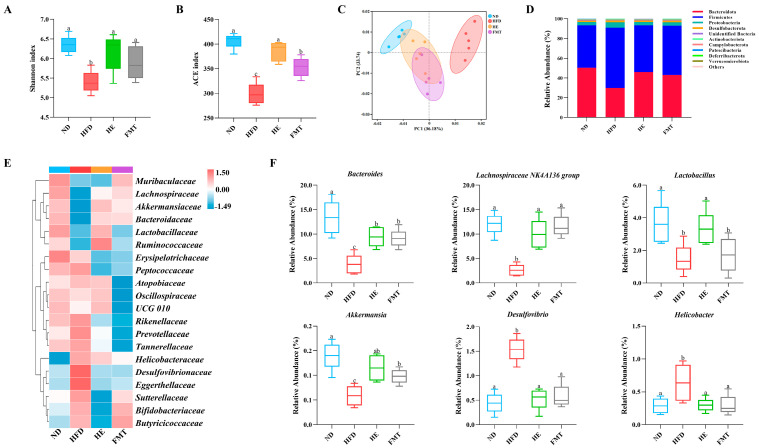
Effects of SCPE and FMT on the gut microbiota in HFD-induced mice. (**A**) Shannon index, (**B**) ACE index, (**C**) PCA scores plot, (**D**) percentage of species at the phylum level, (**E**) heat map of bacterial community relative abundances at the family level, and (**F**) species level differential abundance plot. The different lowercase letters indicate a significant difference (*p* < 0.05) among different groups. The data are presented as the mean ± SEM (*n* = 5).

16S rRNA gene high-throughput sequencing was used to evaluate the effects of SCPE and FMT interventions on HFD-induced gut microbiota dysbiosis. Alpha diversity analysis indicated that the Shannon and ACE indices in the HFD group were notably lower than those in the ND group (Figure 4A,B). However, both SCPE and FMT interventions significantly increased Shannon and ACE indices, indicating that SCPE can enhance the richness and diversity of the gut microbiota. To explore the spatial changes in the microbiota composition among the groups, PCA was performed (Figure 4C). The results revealed a clear separation in the gut microbiota structure between the ND and HFD groups, while the microbiota composition in the HE and FMT intervention groups was closer to that of the ND group, suggesting that SCPE effectively reverses HFD-induced microbial community imbalances.

To further investigate the specific changes in gut microbiota composition following SCPE intervention, we conducted an in-depth analysis of microbial community composition. At the phylum level, the gut microbiota in all groups consisted predominantly of Bacteroidetes, Firmicutes, and Proteobacteria, which together accounted for more than 90% of the total microbial population (Figure 4D). SCPE and FMT treatments mitigated the HFD-induced alterations in gut microbiota composition by decreasing Firmicutes and increasing Bacteroidota levels, thereby lowering the Firmicutes/Bacteroidetes (F/B) ratio. Furthermore, both SCPE and FMT interventions effectively suppressed the HFD-induced overgrowth of Proteobacteria. At the family level, compared to the ND group, HFD markedly decreased the abundance of *Lachnospiraceae*, *Bacteroidaceae*, *Lactobacillaceae*, *Ruminococcaceae*, and *Akkermansiaceae*, while promoting the enrichment of *Desulfovibrionaceae*, *Eggerthellaceae*, and *Helicobacteraceae* (Figure 4E). Notably, SCPE intervention not only significantly suppressed the abnormal proliferation of *Desulfovibrionaceae* and *Eggerthellaceae* but also restored the relative abundance of *Lachnospiraceae*, *Bacteroidaceae*, *Lactobacillaceae*, *Ruminococcaceae*, and *Akkermansiaceae*. Similarly, FMT intervention exhibited comparable regulatory effects, significantly reducing *Desulfovibrionaceae* and *Eggerthellaceae*, while primarily increasing the relative abundance of *Lachnospiraceae*, *Bacteroidaceae*, and *Akkermansiaceae*. Genus-level analysis further revealed the precise regulatory effects of SCPE (Figure 4F). Both SCPE and FMT interventions effectively counteracted the HFD-induced dysbiosis by suppressing pro-inflammatory genera, such as *Desulfovibrio* and *Helicobacter*, and promoting beneficial taxa, including *Bacteroides*, *Lachnospiraceae NK4A136 group*, *Lactobacillus*, and *Akkermansia*.

**Figure 5:**
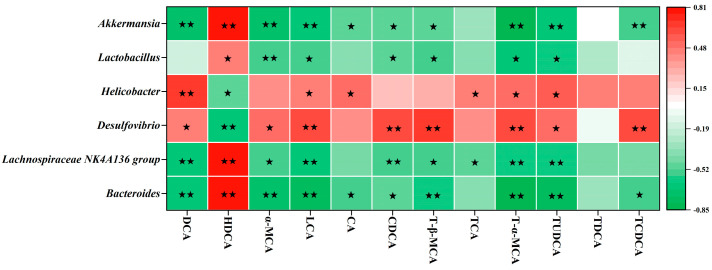
Correlation analysis between characteristic bacteria and fecal BAs in HFD-induced mice. Colors of squares represent the R-value of Spearman’s correlation. ★ and ★★ indicate the significance of association at levels of *p* < 0.05 and *p* < 0.01, respectively.

To further elucidate the potential relationships between gut microbiota and BA metabolism, Spearman correlation analysis was performed on significantly affected genera and fecal BA composition (Figure 5). The results showed that enriched genera (*Bacteroides*, *Lachnospiraceae NK4A136 group*, *Lactobacillus*, and *Akkermansia*) were significantly inversely correlated with multiple BAs, including DCA, α-MCA, LCA, CA, CDCA, T-β-MCA, TCA, T-α-MCA, TUDCA, and TCDCA, but positively correlated with HDCA. Conversely, the significantly reduced genera (*Desulfovibrio* and *Helicobacter*) exhibited the opposite correlation pattern. These findings suggest that SCPE-reshaped gut microbiota may regulate BA metabolism, thereby contributing to its anti-obesity effects.

## 4. Discussion

Obesity has emerged as a global health crisis with complex pathological mechanisms and limited effective interventions. Recently, dietary nutritional strategies for metabolic regulation have garnered significant attention due to their multi-target therapeutic potential. Although previous research has demonstrated the metabolic regulatory effects of SCPE, its specific pathway involvement in lipid metabolism, particularly through the “gut microbiota-BA metabolism” axis, remains insufficiently explored. As a crucial regulator of metabolism, the gut microbiota plays a key role in obesity treatment by modulating BA composition to improve host metabolic function [23]. Through FMT and BA metabolomics, this study is the first to elucidate the molecular mechanism by which SCPE alleviates obesity through gut microbiota remodeling and subsequent regulation of BA metabolism.

In this study, our findings demonstrate that SCPE intervention effectively alleviates HFD-induced obesity and related metabolic dysfunctions. Specifically, SCPE reduced liver, perirenal, and epididymal fat accumulation, improved the histopathological features of liver and epididymal adipose tissues, and lowered both Lee’s index and fasting blood glucose levels. In terms of liver protection, SCPE alleviated oxidative stress by increasing hepatic SOD and GSH-Px activities while reducing MDA levels. Furthermore, serum ALT and AST activities were significantly decreased. It has been reported that liver damage leads to the leakage of AST and ALT from hepatocytes into the bloodstream, resulting in increased serum concentrations [25]. In addition, SCPE effectively regulated lipid metabolism, lowering serum TG, TC, and LDL-C levels while increasing HDL-C levels. Hepatic TG and TC levels were similarly reduced. Notably, the FMT group exhibited key metabolic benefits similar to those observed in the HE group, regulation of body weight, reduction in fat accumulation, and improvements in lipid profiles. These findings suggest that SCPE ameliorates metabolic disorders by modulating gut microbial communities and their metabolites.

Additionally, SCPE intervention significantly regulates BA synthesis and transport by reshaping the gut microbiota. BA biosynthesis occurs through both the classic and alternative pathways, with the classic pathway serving as the primary route, accounting for approximately 75% of BA synthesis [26]. CYP7A1, a key rate-limiting enzyme in the classic pathway, shows a positive correlation with improvements in NAFLD [27], and free fatty acids have been shown to enhance its hepatic expression [28]. The alternative pathway, initiated by CYP27A1, generates 27-hydroxycholesterol as a substrate for subsequent hydroxylation reactions [12]. Ou-Yang et al. [16] reported that upregulating hepatic CYP7A1 and CYP27A1 expression promotes the conversion of cholesterol into BAs, thereby effectively regulating cholesterol metabolism and improving obesity. FXR, the primary BA receptor, modulates glucose homeostasis by enhancing insulin sensitivity and inhibiting hepatic gluconeogenesis, thereby mitigating obesity [29]. Moreover, FXR modulates mucosal barrier function by promoting the growth of SCFA-producing bacteria and regulating immune responses [30]. SHP, acting as a downstream effector of FXR, activates the FXR-SHP signaling pathway, which accelerates lipolysis and reduces lipid accumulation in the liver [31]. Wang et al. [32] found that Simiao Wan reduces intestinal TBA content by upregulating hepatic CYP7A1, FXR, and SHP expression. Furthermore, ASBT inhibitors can modulate signaling in the gut-liver axis, reducing hepatic fat deposition in obese mice [33] and upregulating FXR expression in the liver [34]. ABCG8, a key transporter mediating intestinal cholesterol efflux, directly influences cholesterol absorption efficiency [35]. In this study, FMT intervention significantly enhanced the hepatic expression of FXR, SHP, CYP7A1, and CYP27A1, increased colon ABCG8 expression, and inhibited ASBT expression. These findings suggest that SCPE may regulate the gut microbiota, which in turn affects BA synthesis and transport pathways, ultimately altering BA composition.

Further research suggests that the anti-obesity effects of SCPE are likely attributed to its regulation of gut microbiota-mediated BA composition. BAs serve as important bridges between intestinal microbiota and host metabolism, with changes in their composition directly influencing lipid biosynthesis and cholesterol homeostasis [15,36]. In this study, FMT intervention greatly reduced TBA contents in the serum and feces of HFD mice. Fecal BAs quantification revealed that FMT specifically reduced the contents of unconjugated BAs, including DCA, CA, α-MCA, LCA, and CDCA, as well as conjugated BAs such as T-β-MCA, TCA, TCDCA, TUDCA, and T-α-MCA. Interestingly, the content of HDCA, an unconjugated BA, was notably increased. The reduction of cytotoxic BAs, such as DCA and LCA, likely contributes to metabolic improvements by alleviating gut inflammation and oxidative damage [37]. Xu et al. [38] reported that DCA supplementation induced inflammation in the ileum and colon, altered gut microbiota composition, and led to the accumulation of fecal BAs. Similarly, CA supplementation has been shown to reduce SCFA production, promote intestinal carcinogenesis [39], and enhance the growth of pathogenic microorganisms such as *Erysipelotrichi* [40]. Mouzaki et al. [41] also demonstrated that a marked increase in fecal contents of CA, CDCA, and TBA in individuals diagnosed with non-alcoholic steatohepatitis. T-α-MCA and T-β-MCA are recognized as potent FXR inhibitors, and gut microbiota can suppress BA production through decreasing T-MCA contents and enhancing FXR-mediated *Fgf15* transcription [42]. DCA derivatives, including TUDCA and TCDCA, have been shown to downregulate FXR expression [43]. Additionally, the significant increase in HDCA observed with FMT intervention can promote the growth of beneficial microbial species, such as *Parabacteroides distasonis,* and enhance lipid metabolism by activating PPARα signaling pathways, thereby enhancing hepatic FXR expression [44]. Collectively, these results suggest that SCPE improves obesity by remodeling gut microbiota, which in turn reshapes BA synthesis, transport, and signaling, ultimately restoring metabolic balance.

The gut microbiota is recognized as an “endocrine organ” that regulates BA metabolism through biotransformation processes such as hydrolysis and dihydroxylation [45]. Grau et al. [46] found that gut microbial composition significantly influences BA metabolism and reabsorption efficiency. Prete et al. [47] further revealed that *Lactobacillus plantarum* specifically alters glycol-conjugated BAs, promoting the production of unconjugated BAs such as DCA, CDCA, UDCA, and LCA. Currently, targeting gut microbiota dysbiosis has developed into a promising approach to preventing and treating obesity. Our study found that SCPE effectively enhanced gut microbiota diversity and significantly reduced the F/B ratio, which is typically associated with decreased energy absorption in the host [48]. In addition, BA synthesized by the liver are typically conjugated with glycine or taurine to form bile salts. A reduction in BSH-producing microbes and their enzymatic activity disrupts the transformation from conjugated into unconjugated BAs, ultimately compromising host health [49,50]. In this study, we found a significant elevation in the conjugated/unconjugated BAs ratio in HFD mice, whereas FMT intervention effectively ameliorated this imbalance, suggesting that SCPE may improve BA metabolism by modulating gut microbiota. Further investigation revealed that SCPE notably promoted the proliferation of highly active BSH-producing bacteria, including *Lachnospiraceae*, *Lactobacillaceae*, *Bacteroidaceae*, *Ruminococcaceae*, and *Akkermansiaceae* [51]. At the genus level, SCPE notably increased the abundance of *Bacteroides, Lachnospiraceae NK4A136 group*, *Lactobacillus*, and *Akkermansia*, similar to the findings of Anhe et al. [52]. *Bacteroides* has been shown to regulate symbiotic microbiota and energy metabolism by reducing T-β-MCA and TUDCA contents, while activating the BA-FXR signaling pathway to alleviate insulin resistance and fat accumulation [53,54]. *Lactobacillus* enhances BSH activity, promoting the breakdown of conjugation BAs and reducing lipid absorption [55]. Meanwhile, *Akkermansia* is crucial to maintaining metabolic homeostasis, alleviating obesity, and protecting gut barrier integrity [56]. Notably, FMT intervention also selectively enriched *Bacteroides*, *Lachnospiraceae NK4A136 group*, and *Akkermansia*, further supporting the potential link between SCPE’s anti-obesity properties and gut microbiota remodeling. Moreover, Spearman correlation analysis identified significant relationships between altered gut microbial taxa and the fecal BA profiles. Therefore, SCPE may regulate BA metabolism by reshaping gut microbiota composition, thereby improving cholesterol homeostasis, reducing fat accumulation, and ultimately exerting its anti-obesity effects. Similar regulatory effects on obesity, lipid metabolism, and gut microbiota composition have also been reported in other studies using HFD-fed C57BL/6 mice treated with various plant-derived extracts. For example, apple polyphenol extract alleviated hepatic steatosis by modulating BA synthesis through hepatic FXR signaling, reducing fecal total and primary BA levels, and increasing the abundance of *Akkermansia* [23]. *Lactarius hatsudake* reduced serum total cholesterol, triglycerides, and LDL-C, mitigated liver injury, and remodeled the gut microbiota by increasing *Parabacteroides*, *Muribaculaceae*, and *Oscillibacter* while decreasing *Lachnospiraceae NK4A136 group* [57]. Similarly, blueberry and cranberry anthocyanin extracts significantly reduced body weight gain, improved lipid profiles, and promoted the growth of *Lachnoclostridium*, *Roseburia*, and *Clostridium innocuum group*, leading to increased SCFA production [58]. Fu loose tea and Fu brick tea also prevented obesity and metabolic dysfunction by modulating lipid metabolism–related genes and increasing the abundance of *Akkermansia* and *Turicibacter* [59]. Furthermore, piceatannol, a natural stilbene compound, exerted anti-obesity effects by activating AMPK and inhibiting lipogenic proteins such as C/EBPα, PPARγ, and FAS, while altering gut microbiota composition by increasing *Lactobacillus* and decreasing *Bacteroidetes* [60]. Consistent with these studies, SCPE demonstrated comparable metabolic benefits while representing a unique bee pollen–derived extract with a distinct polyphenolic profile, expanding the current understanding of natural dietary polyphenols that modulate the gut microbiota–BA axis.

However, several limitations should be noted. This study was conducted in a mouse model of HFD-induced obesity, and the translational relevance of these findings to humans remains to be verified. SCPE is a polyphenol-rich extract containing multiple bioactive compounds, but the specific components responsible for the observed metabolic effects have yet to be identified. Although significant correlations were observed among gut microbiota composition, BA metabolism, and metabolic improvement, the causal relationships among these factors require further mechanistic investigation. Future studies, particularly those involving human participants, are warranted to confirm the clinical applicability and safety of SCPE as a functional dietary supplement for obesity management.

## 5. Conclusions

This study elucidates the molecular mechanisms by which SCPE alleviates HFD-induced obesity through modulating the “gut microbiota-BA” metabolism axis. Our findings show that SCPE intervention mitigates obesity by reshaping gut microbiota (enriching BSH-active bacteria), which in turn modulates BA metabolism (improving the BA metabolism-related genes expression, optimizing BA composition), thereby improving cholesterol homeostasis while reducing lipid accumulation. Future studies should focus on identifying the specific bioactive compounds in SCPE and clarifying their interactions with microbial communities and host metabolism. These insights will provide the foundation for developing SCPE as a functional health product.

## Figures and Tables

**Table 1:** TBA content in serum, liver, and feces.

Group	TBA Content (μmol/L)		
	Serum	Liver	Feces
ND	5.50 ± 0.33 ^a^	36.91 ± 1.71 ^a^	46.20 ± 2.14 ^a^
HFD	13.05 ± 1.00 ^b^	43.43 ± 2.39 ^a^	116.77 ± 3.05 ^d^
HE	4.81 ± 0.55 ^a^	36.68 ± 2.46 ^a^	78.84 ± 2.90 ^b^
FMT	6.16 ± 0.32 ^a^	41.27 ± 1.97 ^a^	99.36 ± 2.49 ^c^

The different superscript letters indicate a significant difference (*p* < 0.05) among different groups. The data are presented as the mean ± SEM (*n* ≥ 5).

## Data Availability

The data presented in this study are available from the corresponding author on reasonable request, due to restrictions related to participant privacy.

## Supplementary Materials

The following supporting information can be downloaded at: https://www.mdpi.com/article/10.3390/nu17223597/s1, Figure S1: Effects of SCPE and FMT on food intake in HFD-induced mice; Table S1: Target genes and primers sequence for qRT-PCR.
