# Supplementary material for: Schisandra chinensis Bee Pollen Extract Alleviates Obesity by Modulating Gut Microbiota-Driven Bile Acid Metabolism
Source: Nutrients. 2025 Nov 18;17(22):3597. doi: 10.3390/nu17223597 (PMC12655481; doi:10.3390/nu17223597)
Supplement: Supplementary file 1 [file nutrients-17-03597-s001.zip › nutrients-3957869-supplementary.pdf]

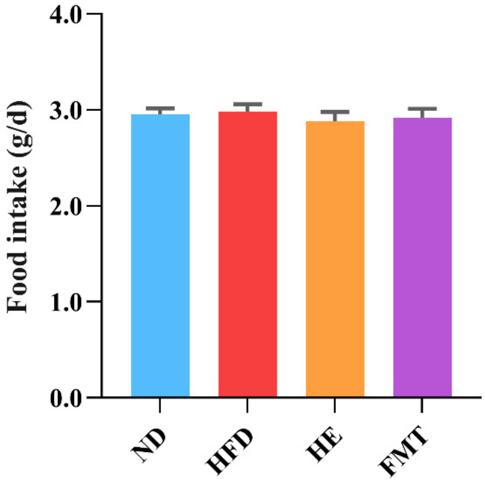

Figure S1. Effects of SCPE and FMT on food intake in HFD-induced mice. The data are presented as the mean ± SEM ($n \geq 5$).

Table S1. Target genes and primers sequence for qRT-PCR.

Gene	Forward primer (5′-3′)	Reverse primer (5′-3′)
FXR	GCTTGATGTGCTACAAAAGCTG	CGTGGTGATGGTTGAATGTCC
SHP	CAGGTCGTCCGACTATTCTGT	AGGCTACTGTCTTGGCTAGGA
ASBT	GTCTGTCCCCCAAATGCAACT	CACCCCATAGAAAACATCACCA
ABCG8	CTGTGGAATGGGACTGTACTTC	GTTGGACTGACCACTGTAGGT
Gapdh	AGGTCGGTGTGAACGGATTTG	GGGGTCGTTGATGGCAACA
